# The evaluation kit for clinical chemistry: a practical guide for the evaluation of methods, instruments and reagent kits

**DOI:** 10.1155/S1463924684000250

**Published:** 1984

**Authors:** G. H. White, C. G. Fraser

**Affiliations:** 1 Department of Clinical Biochemistry Flinders Medical Centre Bedford Park South Australia 5042 Australia; 2 Department of Biochemical Medicine Ninewells Hospital and Medical School Dundee DDI 9SY UK


Journal of Automatic Chemistry, Volume 6, Number 3 (July–September 1984), pages 122–141

### The evaluation kit for clinical chemistry:

### a practical guide for the evaluation of methods, instruments and reagent kits

**G. H. White**
*Department of Clinical Biochemistry, Flinders Medical Centre, Bedford Park, South Australia 5042, Australia*

**and C. G. Fraser**
*Department of Biochemical Medicine, Ninewells Hospital and Medical School, Dundee DD1 9SY, UK*

CONTENTS

SECTION I: *Why evaluate?*
SECTION II: *Terminology*
SECTION III: *The evaluation itinerary*
SECTION IV: *Pre-evaluation assessment*
SECTION V: *Familiarization*
SECTION VI: *Evaluation protocol*
SECTION VII: *Specific studies*
SECTION VIII: *Acceptability criteria*
SECTION IX: *Introduction to service*
SECTION X: *Bibliography*

### Section I: Why evaluate?

The primary role of the clinical chemist is to ensure the provision of diagnostic laboratory services that provide optimal patient care and are as excellent as available technology and local economic factors will allow. However, an acceptable service is provided only if high standards of analytical performance are achieved and maintained. To monitor analytical performance, most laboratories use comprehensive internal and external quality control and assurance programmes. It should be realized, however, that the strategy of these schemes is to compare present performance with previous performance and not to provide data on whether the method itself is capable of fulfilling the analytical and clinical performance standards required.

Thus, before a method is brought into routine service in any laboratory, even the smallest, it is necessary to objectively assess the method itself, not only in terms of its analytical characteristics and ability to fulfill clinical needs, but also its impact on the laboratory, staff and budget. To obtain the data that allow a method to be so fully defined means that a proper and full evaluation must be carried out. It is preferable to do such an evaluation before a method (whether an instrument or reagent kit set) is purchased, but methods that are already purchased, even if in use for some time, should also be subjected to the same close scrutiny of evaluation. Once the evaluation has been made, the method is fully characterized. The data generated can then be retained in the laboratory; only then can quality control and assurance programmes be instituted and used to ensure that the well-defined and acceptable analytical performance of the method is being maintained .

The total evaluation process should be a logical progression through the steps of pre-purchase or pre-service assessment, familiarization, evaluation and objective assessment of acceptability. None of these steps should be omitted before a candidate method is brought into routine service.

Many evaluation protocols have been published in the literature of clinical chemistry. Most of these are well thought-out and carefully written. They tend, however, to be written for the expert and are consequently somewhat theoretical. A brief working guide for the clinical chemistry 'layman' has until now been lacking. This publication provides such a practical guide—a route-map for the driver who has either never been on this road before or is not very familiar with the terrain.

The 'Evaluation Kit' is simple to use. Section II contains the terms and phrases used in evaluation theory and practice; this may be omitted by those familiar with field. Sections III to IX provide the detailed route-map, starting with the itinerary, and then progressing in a logical manner through the stages of the evaluation process.

This material is based on practical experience, on theory, on previously published work and on current national and international recommendations. Major sources of information are acknowledged in the Bibliography (Section X).

### Section II: Terminology

Many of the definitions of the terms and phrases used in clinical chemistry are yet to be agreed upon universally. In this publication, the definitions promulgated by the International Federation of Clinical Chemistry are generally used.

| | |
|---|---|
| *Aliquot:* | A measured portion of a whole having the same composition, |
| *Analyte:* | The component to be measured. |
| *Analytical range:* | The range of concentration (or other quantity) in the specimen over which the method is applicable without modification. |
| *Assigned value:* | Value assigned either arbitrarily or from preliminary evidence. |
| *Bias:* | The same as inaccuracy. |
| *Batch:* | The same as run. |

*Calibration:* The procedure of relating a reading to the quantity required to be measured.
*Carry-over:* The influence of a sample on a following one.
*Comparative method:* A method to which the test method is compared. The characteristics of such a method should be known.
*Consensus value:* Value derived from a set of results.
*Control material:* Material. used for quality control purposes.
*Definitive method:* A method which after exhaustive investigation has been shown to have no known source of inaccuracy or ambiguity.
*Detection limit:* The smallest single result which, with a stated probability, can be distinguished from a true blank.
*Error:* Difference between an estimate of a quantity and its true value.
*External quality assurance:* Procedure of utilizing, for quality control purposes, the results of several laboratories which analyse the same specimens.
*Imprecision:* Standard deviation or coefficient of variation of the results in a set of replicate experiments.
*Inaccuracy:* Numerical difference between the mean of a set of replicate measurements and the true value.
*Interference:* The effect of a component, which does not by itself produce a reading, on the accuracy of measurement of another component.
*Matrix:* The nature of the sample itself.
*Primary standard:* A primary standard solution is used as a calibration standard in which the concentration is determined solely by dissolving a weighed amount of a substance, of known chemical composition and sufficient purity (a primary standard material), in an appropriate solvent and making to a stated volume or weight.
*Reading:* The value indicated on the scale of an instrument or analytical device.
*Reagent kit set:* Two or more different clinical or general laboratory materials (excluding reconstituting materials), with or without other components, packaged together, and designed for the performance of a procedure for which directions are supplied with the package.
*Recovery:* The ability of an analytical method to estimate pure analyte added to the sample.
*Reference method:* A method which after exhaustive investigation has been shown to have negligible inaccuracy in comparison to its imprecision.
*Reference interval:* A range of values with which an observed value is compared for interpretative purposes.
*Replicate:* Analysis of the same sample a number of times.
*Result:* Final value obtained for a measured quantity after performing a measuring procedure, including all sub-procedures and laboratory evaluations.
*Run:* A set of consecutive assays performed without interruption. The results are usually calculated from the set of calibration standard reading.
*Sample:* The appropriately representative part of a specimen which is used in the analysis.
*Specificity:* The ability of a method to determine solely the component(s) it purports to measure.
*Specimen:* The material available for analysis.
*Standard:* Material or solution with which the sample is compared in order to determine the concentration (or other quantity).
*Stated value:* A value stated without official certification.
*True value:* The correct concentration (or other quantity).

The following statistical terms are used in the 'Evaluation Kit':

*Coefficient of variation (CV):* Relative standard deviation, the standard deviation expressed as a fraction of the mean.
*Correlation coefficient (r):* A statistic which estimates the degree of association between two variables.
*F-test:* A statistical test in which the differences between two variances (squares of standard deviations) is tested for significance. The test investigates the hypothesis that there is no difference between the two variances.
*Intercept:* The place at which a line on a graphical plot intersects an axis.
*Linear regression analysis:* A technique for estimation of the best linear relationship between two variables.
*Mean:* The arithmetic average of a set of data.
*Outliers:* Values which do not agree with the majority of values.
*Slope:* The angle of a line on a graph expressed as a ratio of $y$-units to $x$-units.
*Standard deviation:* A statistic which describes the dispersion of a set of values about the mean.
*t-Test:* A statistical test used to assess the difference between means.

### Section III: The evaluation itinerary

There are six stages in any evaluation:

(1) Pre-evaluation assessment.
(2) Familiarization.
(3) Evaluation.
(4) Specific studies.
(5) Assessment of performance.
(6) Introduction to routine service.

The rationale of each of these necessary stages is briefly outlined in the following.

### *(1) Pre-evaluation assessment*

The objective evaluation that must be performed on an instrument or reagent kit set before it is purchased and introduced into routine laboratory service uses both materials and time. Thus, it is generally not sensible or cost-effective to fully assess *all* the available commercial options (candidate methods) in the laboratory. It is therefore necessary to make a pre-selection of preferably not more than three options, these being the candidates that appear to be the most serious contenders for the final choice. Such a pre-selection is often based on no more than a superficial impression of potential suitability that may have been gained from commercial advertising, published evaluations or the previous experience of colleagues. The pre-selection problem may be simplified if there are few commercially available candidates.

The one, two or three likely candidates are first subjected to a *pre-evaluation assessment*, the object of which is to determine whether the candidate is likely to:

(*a*) Perform all or part of the clinical task required.
(*b*) Harmonize with the current laboratory organization and philosophy.
(*c*) Be economic.
(*d*) Not become quickly out-dated.

Completion of the pre-evaluation assessment should allow an objective selection of a single candidate that fulfills sufficient criteria to warrant laboratory evaluation.

### (2) *Familiarization*

When a candidate becomes the *test method* (that is, it has arrived at the laboratory either as a firm purchase or on loan for evaluation) it should, if possible, be located and used at the site selected for its planned routine use. The use of a separate evaluation area and specialist staff may not reveal problems that could arise when the test method is introduced into the routine laboratory.

Once installed, the test method must undergo a *familiarization period*, during which time the instrument or reagent kit set is generally examined and used according to the manufacturer's instructions. Prior to commencing the familiarization period, steps must be taken to ensure that the staff who are to perform the evaluation are well-trained and competent, and that all basic laboratory equipment, such as pipettes, balances and waterbaths, which are ancillary requirements for the technique, are satisfactory. During the familiarization period, a general impression of the following points should be gained:

Is the test method:

(*a*) Doing more or less what was expected?
(*b*) More difficult to operate than expected?
(*c*) Showing no major problems with siting, services, or safety?
(*d*) Acceptable to staff?
(*e*) Acceptable to the manufacturer's representatives, if the method has been provided for trial?

At the conclusion of the familiarization period, decisions are made as to whether the test method is likely to fulfill the needs of the laboratory and whether a formal evaluation should be initiated.

### (3) *Evaluation*

A newly launched instrument or reagent kit set may be accompanied by an instruction manual that includes brief and selected details of the performance obtained by the manufacturer. Such data have been generally obtained under ideal conditions, either in the research and development laboratories of the manufacturer or at one or two selected external centres. The performance details quoted are only a guide and do not guarantee similar results in the working laboratory.

If an independent evaluation of the test method using accepted criteria is unavailable as a paper in a reputable journal then a *full evaluation* must be carried out by the laboratory before a decision on acceptance can be taken and subsequent introduction to service initiated.

If the test method has been in general routine use for some time, it is likely that one or more independent and full evaluations will have been published; these are usually readily obtainable. The steps and criteria used in the evaluation should be checked against those recommended in one or more authorative references (see Bibliography in Section X). If the evaluation report appears to be satisfactory, it is necessary only to ensure that the test method will perform in a similar or satisfactory way in the laboratory. In this case a modified or *short evaluation* provides adequate information for an informed decision to be made as to suitability.

### (4) *Specific studies*

Evaluation protocols are designed to elicit (from any analytical system) the basic data that allow the general performance characteristics of that method to be defined. However, any evaluation protocol is unavoidably general in its approach and therefore *specific studies* that explore the unique properties of the method under consideration may have to be designed and executed in the laboratory. Relevant protocols are often available in the literature.

Disappointing or poor evaluation results should not lead to an automatic rejection of the test method. Instead, specific studies should be carried out in an attempt to identify whether one or more components of the test method, or associated in-house equipment, are making a significant contribution to the unacceptable performance.

Specific studies are also intended to reveal any factors that, in the future, may compromise the performance of the method during the evaluation. The performance characteristics themselves may also be improved by use of knowledge gained in these special studies. Since the range of methods in current use is wide, it will be possible for this publication to provide only an indication of the areas in which specific studies would be of major benefit.

### (5) *Assessment of performance*

Having produced a large quantity of objective information during a short or full evaluation, it is then necessary to decide whether the data indicate that the test method can undertake the alloted task(s) in the routine service laboratory. This is the crucial decision that may lead to a successful installation or an expensive failure. Objective criteria must be strictly applied at this stage.

After short or full evaluation, a final decision can be taken on the 'where, how and when' of the role of the test method in the routine service of the laboratory.

### (6) *Introduction to routine service*

The ease with which the new method is slotted into the routine work of the laboratory depends both on its complexity and on whether it was evaluated at its future work-site by the routine staff or elsewhere by staff that specialize in evaluation. This last stage of bringing a new method on-line is concerned with such points as:

(*a*) Are all staff who are liable to use the method adequately trained?
(*b*) Are trouble-shooting and maintenance schedules prepared?

(*c*) Are adequate reagents and spares available?
(*d*) Are adequate quality control data and internal quality control and external quality assurance programmes available?
(*e*) Are laboratory users aware of any consequent change in specimen requirements or reference intervals?
(*f*) Have specific studies, or action required, been taken to ensure that the evaluation performance characteristics will prevail for all specimens and conditions that may be encountered by the new method in the future?
(*g*) Is the manufacturer, or his agent, satisfied with the performance of the instrument or method?

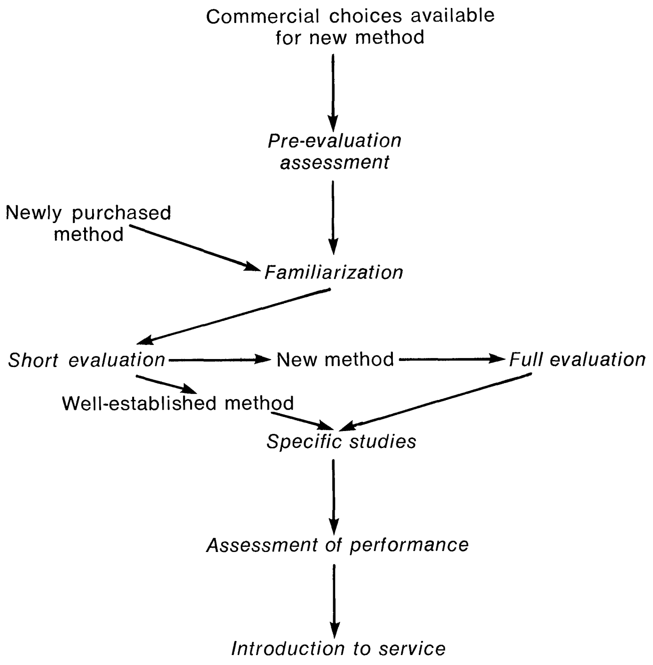

*Figure 1. The evaluation itinerary.*

### Section IV: Pre-evaluation assessment

### *Aims*

(1) To define the function, performance characteristics and other factors that are ideally required by the laboratory for its new method.
(2) To identify the candidate method that is most likely to fulfill the total specification.

The assessment of the candidates should be made using the detailed check-list in figure 2.

### *Notes on the check-list (figure 2)*

*(1) Candidates*

Ideally no more than three candidate instruments or reagent kit sets should be considered. The primary selection is often made on impressions gained from commercial advertising or exhibitions, or on performance reports from colleagues, or the literature. Record brief identifying details of the candidates that have been selected.

*(2) Analytical features*

(*a*) Analytes: list the analyte estimations required by the laboratory and those that are offered by each candidate.
(*b*) Analytical flexibility: only performs full range of analytes offered = FULL;
performs full analyte range, but suppresses unwanted test results = SEMI;
performs only tests selected from analytes offered = SELECT.
Record combination of tests required and that offered by each candidate.
(*c*) Patient type.
Record: adult, child, infant, neonate.
(*d*) Sample type.
Record: blood, plasma, serum, urine, CSF, synovial, pleural, other.
(*e*) Sample volume:
Record: sample volume(s) required and used.

*(3) Service needs*

(*a*) Routine/emergency.
Record whether analyses are required and can be performed either in routine (R), emergency (E), or both situations (R + E).
(*b*) Immediate emergency mode.
Record ability during a routine run to analyse an emergency specimen as the next specimen without compromising the routine run or specimen identification.

*(4) Analytical performance*

(*a*) Published evaluation.
Record whether partial or full evaluation(s) are available, and references.
(*b*) Year candidate method introduced.
Record year when instrument or reagent kit set first became commercially available.
(*c*) Standards or mode of calibration.
Record whether standards, primary or secondary, or calibration factors are used.
(*d*) Assignment of standard value.
Record mode that has been used to assign value to standard. For example, the standards provided with radioimmunoassay kits for peptides and proteins are generally calibrated against various international preparations or primary standards (World Health Organization, Medical Research Council, etc.).
(*e*) Reference intervals.
Record current reference intervals used by laboratory and those quoted by manufacturer.
(*f*) Throughput time.
1 sample (ready): the time required to produce a single patient result when the method is already prepared for immediate operation (include standards and appropriate quality control).
Instruments: warmed up and calibrated.
Reagent kit sets: reagents already prepared.
1 sample (cold): the time required to provide a patient result when the method is in an unprepared state.
Rate: the number of samples (test, standard or quality control) that can be processed in a selected time period, for example instruments: $x$ samples/hour if the rate of analysis can be maintained each hour; reagent kit sets: $x$ samples/two days may be typical of a lengthy radio-immunoassay method.
(*g*) Between-day imprecision (SD or CV).
Sales literature normally provides some basic performance data, usually both between-day and within-

*Figure 2. Pre-evaluation assessment.*

| | Laboratory requirement | Candidates 1 | Candidates 2 | Candidates 3 |
|---|---|---|---|---|
| (1) *Model/name* | | | | |
| Manufacturer | — | | | |
| Sample available for evaluation | — | | | |
| Laboratory space required | — | | | |
| (2) *Analytical features* | | | | |
| (*a*) Analytes | | | | |
| (*b*) Analyte flexibility | | | | |
| (*c*) Patient type | | | | |
| (*d*) Sample type | | | | |
| (*e*) Sample volume | | | | |
| Fixed | ...μl/ml | ...μl/ml | ...μl/ml | ...μl/ml |
| Various | .., .., ...μl/ml | .., .., ...μl/ml | .., .., ...μl/ml | .., .., ...μl/ml |
| Variable range | ..–...μl/ml | ..–...μl/ml | ..–...μl/ml | ..–...μl/ml |
| (*f*) Maximum batch size per run | | | | |
| (*g*) Units of test results | | | | |
| Comments: | | | | |
| (3) *Service needs* | | | | |
| (*a*) Routine, emergency | R, E, R+E | R, E, R+E | R, E, R+E | R, E, R+E |
| (*b*) Immediate emergency mode | N Y | N Y | N Y | N Y |
| Comments: | | | | |
| (4) *Analytical performance* | | | | |
| (*a*) Published evaluation | — | N Y | N Y | N Y |
| References | | | | |
| (*b*) Year candidate method introduced | — | 19.. | 19.. | 19.. |
| (*c*) Standards or calibrators supplied | — | N Y | N Y | N Y |
| (*d*) Assignation of standard value | | | | |
| (*e*) Manufacturer's suggested reference interval | Present interval: ..–... | ..–... | ..–... | ..–... |
| (*f*) Throughput time for: | | | | |
| 1 sample (ready) | | | | |
| 1 sample (cold) | | | | |
| rate (routine) | | | | |
| (*g*) Between-day imprecision | Present method: | *a* *b* | *a* *b* | *a* *b* |
| (CV%) Low | % | | | |
| Med. | % | | | |
| High | % | | | |
| (*a*) Manufacturer's data | | | | |
| (*b*) Published evaluation | | | | |
| Comments: | | | | |
| (5) *Staff factors* | | | | |
| (*a*) Operator skill (routine/out-of-hours) | | | | |
| (*b*) Additional staff | — | Y N | Y N | Y N |
| (*c*) Training of present staff | — | Y N | Y N | Y N |
| (*d*) Safety hazards | — | Y N | Y N | Y N |
| Comments: | | | | |

*continued —*

run imprecision for analyses of quality control materials. These values should be recorded and compared with the published data, if available. Care should be taken in noting whether the data were derived from a meaningful number of experimental measurements at relevant levels of analyte in an appropriate matrix.

(5) *Staff factors*

(*a*) Operator skill required.

Record whether satisfactory operation is possible by: untrained new staff, experienced technician, graduate, other (both for routine and out-of-hours services).

(*b*) Additional staff needed.

Record whether additional staff will be required.

(*c*) Training of present staff.

Record whether training of staff will require significant time.

(*d*) Safety hazards.

Record potential hazards, for example radioisotopes, corrosive chemicals, potential carcinogens.

(6) *Laboratory supplies*

Record:

(*a*) Whether the laboratory requires reagents to be supplied ready-made and whether the manufacturer is able to supply these. Record alternative suppliers and note their previous record of reliability.

*Figure 2. Pre-evaluation assessment (continued).*

| | Laboratory requirement | Candidates 1 | Candidates 2 | Candidates 3 |
|---|---|---|---|---|
| (6) *Laboratory supplies* | | | | |
| (*a*) Reagents supplied | Y N | Y N | Y N | Y N |
| (*b*) Reagent formulae provided | Y N | Y N | Y N | Y N |
| (*c*) Disposable items required | — | | | |
| (i) | | | | |
| (ii) | | | | |
| (iii) | | | | |
| (iv) | | | | |
| (v) | | | | |
| (*d*) Additional equipment required | — | Y N | Y N | Y N |
| (i) Available and compatible | — | Y N | Y N | Y N |
| (*e*) Additional equipment requiring purchase | — | Y N | Y N | Y N |
| Nature and approximate cost | | | | |
| (*f*) Built-in fault diagnosis | Y N | Y N | Y N | Y N |
| (*g*) Capable of in-house routine maintenance | Y N | Y N | Y N | Y N |
| (*h*) Local manufacturer-trained engineer/agent | Y N | Y N | Y N | Y N |
| (*i*) Spares always stocked by local agent | — | Y N | Y N | Y N |
| Comments: | | | | |
| (7) *Services* | | | | |
| (*a*) Overall dimensions | ...×...×... | ...×...×... | ...×...×... | ...×...×... |
| (*b*) Structural changes to laboratory | — | Y N | Y N | Y N |
| (*c*) Adequate facilities already available for: | | | | |
| Electricity | — | Y N | Y N | Y N |
| Water | — | Y N | Y N | Y N |
| Drainage | — | Y N | Y N | Y N |
| Ventilation | — | Y N | Y N | Y N |
| Solvent disposal | — | Y N | Y N | Y N |
| Radiation disposal | — | Y N | Y N | Y N |
| Comments: | | | | |
| (8) *Economics* | | | | |
| (*a*) Capital cost | Budget: ...... | | | |
| (*b*) Working life of method | — | | | |
| (*c*) Cost per sample | Present method: | | | |
| Cost per run (reagents and disposables only) | | | | |
| (*d*) Annual cost of routine maintenance spare parts | | | | |
| (*e*) Cost of annual maintenance contract | | | | |
| Comments: | | | | |
| (9) *Decision* | | | | |
| Number of features compatible with laboratory requirement | — | | | |
| (10) Order of preference | | | | |
| Comments: | | | | |

*continued*

(*b*) User preference to produce reagents if the formulae are provided.

(*c*) Disposable items such as pipette tips, tubes or vials that have to be supplied by the user.

(*d*) Equipment to be provided by the laboratory, for example dispensers, centrifuge, water-bath, spectrophotometer (note wavelength requirement).

(*e–f*) Other materials, spares and maintenance and service expertise supplied, or potentially supplied, by the manufacturer and/or required by the laboratory are recorded in the appropriate sections of the check-list.

(7) *Services*

The work space, laboratory structural changes and services required are recorded in the appropriate sections of the check-list.

(8) *Economics*

The capital cost, period of amortization and likely running costs are recorded appropriately.

(9) *Decision*

A pre-evaluation assessment cannot provide a decision from a simple generation and inspection of the final score reached by each candidate. It does, however, allow a rapid identification and collation of the method features that are important and desirable to the laboratory and also identifies how far each candidate satisfies or falls short of the ideal. After this assessment has been performed, it should be possible to rank the candidates in order of preference and make a decision on whether the first choice is sufficiently suitable to warrant an example being obtained and evaluated in the laboratory (*the test method*).

### Section V: Familiarization

The aim of the familiarization period is to ensure, as far as possible, that the test method selected from the candidates will be evaluated under conditions that:

(1) Do not compromise the potential of the method.
(2) Represent the ideal laboratory conditions that would prevail for routine use.

After installation of the test method in the laboratory and checking the performance of all ancillary equipment required, the staff performing the initial study should accustom themselves to the preparation of reagents, samples and the basic operations of the system. When confident, the operator(s) should analyse a small number of samples, preferably using quality control and patient samples with known values. During the period, the *familiarization check-list* (figure 3) is completed.

The check-list is mainly self-explanatory: the following brief notes are provided to assist the evaluators:

(1) If the test method is first set up and run in a special section for evaluation, particularly if staffed by individuals who will not be the routine users, then the evaluators should attempt to delineate potential problems that may arise when the test method is transferred to the routine location, for example space requirements, service facilities, staff skills and special techniques.
(5) If the components provided by the manufacturer (for example tubes) are not compatible with existing equipment (for example gamma counter), or are less than satisfactory (for example having to use a 200 μl and a 100 μl pipette to dispense 300 μl), steps to rectify the deficiency should be taken before proceeding to an evaluation.

### Section VI: Evaluation protocol

### *(1) Inaccuracy and within-run imprecision*

Inaccuracy and within-run imprecision can be assessed by various experimental approaches. The experimental scheme detailed here is particularly useful when only brief assessments of inaccuracy and imprecision are required; in addition, the results also provide information on linearity and may indicate potential interferences. The following experimental procedure should be carried out to provide a *short evaluation.*

*Procedure*

Analyse a number of samples obtained from specimens from patients (at least 40 and preferably 100) *in duplicate* by the test method. The sample duplicates should be within the same analytical batch, but should be randomly distributed throughout the batch.

The samples should be carefully selected, if possible, from specimens that have been analysed by the currently used method, so that the levels of analyte span the analytical range of the method. Lipaemic, icteric and haemolysed specimens should be included.

Analyse the samples in duplicate by a comparative method. The comparative method should be selected with care. Ideally, it should be a method that has no inaccuracy and small imprecision, which infers that a reference method or definitive method should be used. Such methods are either unavailable (for example isotope dilution mass spectrometry) or impracticable. Therefore the usual practice is to use either the best method that the laboratory has available or a routine method whose bias and imprecision are known from, for example, internal and external quality control and assurance data.

*Figure 3. Familiarization check-list.*

| | Candidate | | Action required prior to evaluation |
|---|---|---|---|
| | YES | NO | |
| (1) Method located at site planned for routine use | □ | □ | |
| (2) Services satisfactory: Power | □ | □ | |
| Water | □ | □ | |
| Drainage | □ | □ | |
| Ventilation | □ | □ | |
| Solvent disposal | □ | □ | |
| Radiation disposal | □ | □ | |
| (3) (*a*) Instruction manual satisfactory | □ | □ | |
| (*b*) Need to condense/alter instructions for routine use | □ | □ | |
| (4) Problems with reagent preparation/reconstitution | □ | □ | |
| (5) Method components compatible with in-house equipment | □ | □ | |
| (6) Method appears to function according to manufacturer's expectations | □ | □ | |
| (7) Staff confident of basic operations of method | □ | □ | |
| (8) Approximate time for staff to be confident of usage | | | |
| (9) Satisfactory results obtained from trial analyses | □ | □ | |
| (10) Safety hazards | □ | □ | |
| (11) Unexpected problems | □ | □ | |
| (12) Sufficient reagents/chart paper/quality-control materials to proceed with *Short* or *Full evaluation* | □ | □ | |
| (13) Manufacturer satisfied with results of familiarization period | □ | □ | |

Ideally, the same standards or calibration materials should be used for both test and comparative methods. Single batches of reagents should be used throughout for both methods. Where reagents are unstable, they should be prepared as required from the same lot of chemicals.

The results of analysis are recorded in columns A and B (for the test method) and C and D (for comparative method) in figure 4. The other columns may be used for the calculations as described below. At this point, note the characteristics of both test and comparative methods in figure 4(*b*).

*Graphical analysis*

Plot the *first* result obtained for each sample by the test method (A) against the *first* result obtained by the comparative method (C) on a graph. Both scales should be chosen to accommodate a range from zero to the highest result generated.

Before proceeding with statistical data analysis, *outliers should be excluded* at this stage. Potential outliers are points which do not lie in the main cluster of points plotted. Sample duplicate results (B for test method and D for comparative method) should be inspected to check whether a gross blunder, for example a sample mix-up, has taken place. To identify outliers by statistical methods is extremely complex, and therefore a simple visual inspection of data points is recommended. If a point is distant from the main cluster and its duplicate would lie with the main body of points, then the rogue point can be rejected. Outliers can also be rejected if there are logical reasons for data discrepancies (for example if a manipulative error occurred).

Linearity should also be assessed by visual inspection. Subsequent statistical analysis is carried out on only those results that fall *within the linear range* of the test method.

*Data analysis*

Calculate the mean, standard deviation and coefficient of variation for the results obtained by both the test and comparative methods.

*If calculation facilities are unavailable:*

(1) *Add all the results in column A ($\Sigma x$) and divide the sum by the number of analyses ($n$) to give the mean result ($\bar{x}$) for the test method. Repeat for column C (comparative method).*

(2) *For each pair, calculate, by subtraction, the difference between the paired results (results in column A—results in column B for the test method, and results in column C—results in column D for the comparative method) and record in the next column the numerical value of the difference ($d$), disregarding the sign ($\pm$).*

(3) *Square these differences and record in the next column ($d^2$).*

(4) *Add the resultant values, divide these totals ($\Sigma d^2$) by twice the number of pairs of samples analysed ($2n$), and take the square roots of these numbers to obtain the standard deviation for both test and comparative methods.*

(5) *Calculate the coefficients for the test and comparative method from the formula:*

$$CV = \frac{SD}{\bar{x}}.$$

It is recommended that, since the imprecision may depend on the level of analyte, the results be divided into three groups (low, medium and high results), if there are at least 20 pairs of results in each group. The number of pairs, means, standard deviations and coefficients or variation for each group are calculated as detailed above and recorded in the figures 4(*a*) and 4(*b*).

*F-test*

In order to compare the imprecisions of test and comparative methods, the F-test is used. F is calculated from the ratio of the square of the *larger* standard deviation to the square of the *smaller* standard deviation. The calculated F should be recorded, compared to tabulated values for the appropriate number of duplicate analyses, and the significance assessed at various levels of probability (usually 95% and 99%) (see appropriate references in the Bibliography in section X).

*'t'-test*

The means of the sets of results obtained by test and comparative methods should be compared using the *t*-test.

*If calculation facilities are unavailable:*

(1) *Derive $S^2$*

$$S^2 = \frac{[(n_T - 1)S_T^2 + (n_C - 1)S_C^2]}{(n_T + n_C - 2)}$$

*where $n_T$ is the number of analyses performed by the test method; $n_C$ is the number of analyses performed by the comparative method; $S_T^2$ is the square of the standard deviation for the test method; and $S_C^2$ is the square of the standard deviation for the comparative method.*

(2) *Calculate S, the square root of $S^2$.*

(3) *Derive t from the formula:*

$$t = \frac{(\bar{x}_T - \bar{x}_C)}{S} \cdot \sqrt{\frac{n_T \cdot n_C}{n_T + n_C}}$$

*where $x_T$ and $x_C$ are the means of the results generated from the test and comparative methods respectively.*

Record the value for *t*. *t*-Tables should then be consulted, at the appropriate number of degrees of freedom ($n_T + n_C - 2$), to assess the probability that there is no significant difference between the two means (usually at 95% or 99% levels) (see Bibliography in section X).

Linear regression analysis should be performed and the correlation coefficient, slope, intercept, and, ideally, standard deviations of slope and intercept should be calculated and recorded.

*If calculation facilities are unavailable:*

(*a*) *For the results obtained by the test method, note the number ($n_T$), and the sum of all results ($\Sigma x_T$), and calculate the mean*

$$\frac{(\Sigma x_T)}{n_T}.$$

(*b*) *Calculate the sum of the squares of the test results $\Sigma x_T^2$.*

(*c*) *Calculate the square of the sum of the test results $(\Sigma x_T)^2$.*

(*d*) *Calculate*

$$\Sigma x_T^2 - \frac{(\Sigma x_T)^2}{n}$$

*which is equal to $\Sigma(x_T - \bar{x}_T)^2$.*

(*e*) *Follow the above procedures for the comparative method to derive*

$$\Sigma x_C^2 - \frac{(x_C)^2}{n}$$

*which is equal to $\Sigma(x_C - \bar{x}_C)^2$.*

(*f*) *Multiply the two figures derived in Steps* (*d*) *and* (*e*) *and take the square root to give*

$$\sqrt{\Sigma(x_T-\bar{x}_T)^2\cdot\Sigma(x_C-\bar{x}_C)^2}.$$

(*g*) *Multiply each value of* $x_T$ *by the corresponding value for* $x_C$ *and then add all the values together to give* $\Sigma x_T\cdot x_C$.

(*h*) *Multiply the sum of all the results for* $x_T(\Sigma x_T)$ *by the sum of all the results for* $x_C(\Sigma x_C)$ *and divide by the number of pairs of observations to give*

$$\frac{\Sigma x_T\cdot\Sigma x_C}{n}.$$

(*i*) *Subtract the number obtained from the number obtained in Step* (*g*) *to give*

$$\Sigma x_T\cdot x_C-\frac{\Sigma x_T\cdot x_C}{n}.$$

(*j*) *Calculate r by dividing the number generated in Step* (*i*) *by the number obtained in Step* (*f*)

$$r=\frac{\Sigma x_T\cdot x_C-\dfrac{\Sigma x_T\cdot\Sigma x_C}{n}}{\sqrt{\Sigma(x_T-\bar{x}_T)^2\cdot\Sigma(x_C-\bar{x}_C)^2}}.$$

(*k*) *Calculate the regression equation*

$$x_T=a\cdot x_C+b$$

*where*

$$a=\frac{\Sigma(x_T-\bar{x}_T)(x_C-\bar{x}_C)}{\Sigma(x_T-\bar{x}_T)^2}$$

*and*

$$b=\bar{x}_T-a\cdot\bar{x}_C.$$

*Summary*

At this stage, a brief summary of the results of this short evaluation should have been collated and recorded; also record

*Figure 4*(*a*). *Inaccuracy and imprecision.*

| | *Test method* | | | | *Comparative method* | | | |
|---|---|---|---|---|---|---|---|---|
| *No.* | A Result | B Result | d | $d^2$ | C Result | D Result | d | $d^2$ |
| 1 | | | | | | | | |
| 2 | | | | | | | | |
| 3 | | | | | | | | |
| 4 | | | | | | | | |
| 5 | | | | | | | | |
| 6 | | | | | | | | |
| 7 | | | | | | | | |
| 8 | | | | | | | | |
| 9 | | | | | | | | |
| 10 | | | | | | | | |
| 11 | | | | | | | | |
| 12 | | | | | | | | |
| 13 | | | | | | | | |
| 14 | | | | | | | | |
| 15 | | | | | | | | |
| 16 | | | | | | | | |
| 17 | | | | | | | | |
| 18 | | | | | | | | |
| 19 | | | | | | | | |
| 20 | | | | | | | | |
| 21 | | | | | | | | |
| 22 | | | | | | | | |
| 23 | | | | | | | | |
| 24 | | | | | | | | |
| 25 | | | | | | | | |
| 26 | | | | | | | | |
| 27 | | | | | | | | |
| 28 | | | | | | | | |
| 29 | | | | | | | | |
| 30 | | | | | | | | |
| 31 | | | | | | | | |
| 32 | | | | | | | | |
| 33 | | | | | | | | |
| 34 | | | | | | | | |
| 35 | | | | | | | | |
| 36 | | | | | | | | |
| 37 | | | | | | | | |
| 38 | | | | | | | | |
| 39 | | | | | | | | |
| 40 | | | | | | | | |
| 41 | | | | | | | | |
| 42 | | | | | | | | |
| 43 | | | | | | | | |
| 44 | | | | | | | | |
| 45 | | | | | | | | |
| 46 | | | | | | | | |
| 47 | | | | | | | | |
| 48 | | | | | | | | |
| 49 | | | | | | | | |
| 50 | | | | | | | | |

*continued —*

*Figure 4(a). Inaccuracy and imprecision (continued).*

| | Test method | | | | Comparative method | | | |
|---|---|---|---|---|---|---|---|---|
| *No.* | A | B | d | $d^2$ | C | D | d | $d^2$ |
| 51 | | | | | | | | |
| 52 | | | | | | | | |
| 53 | | | | | | | | |
| 54 | | | | | | | | |
| 55 | | | | | | | | |
| 56 | | | | | | | | |
| 57 | | | | | | | | |
| 58 | | | | | | | | |
| 59 | | | | | | | | |
| 60 | | | | | | | | |
| 61 | | | | | | | | |
| 62 | | | | | | | | |
| 63 | | | | | | | | |
| 64 | | | | | | | | |
| 65 | | | | | | | | |
| 66 | | | | | | | | |
| 67 | | | | | | | | |
| 68 | | | | | | | | |
| 69 | | | | | | | | |
| 70 | | | | | | | | |
| 71 | | | | | | | | |
| 72 | | | | | | | | |
| 73 | | | | | | | | |
| 74 | | | | | | | | |
| 75 | | | | | | | | |
| 76 | | | | | | | | |
| 77 | | | | | | | | |
| 78 | | | | | | | | |
| 79 | | | | | | | | |
| 80 | | | | | | | | |
| 81 | | | | | | | | |
| 82 | | | | | | | | |
| 83 | | | | | | | | |
| 84 | | | | | | | | |
| 85 | | | | | | | | |
| 86 | | | | | | | | |
| 87 | | | | | | | | |
| 88 | | | | | | | | |
| 89 | | | | | | | | |
| 90 | | | | | | | | |
| 91 | | | | | | | | |
| 92 | | | | | | | | |
| 93 | | | | | | | | |
| 94 | | | | | | | | |
| 95 | | | | | | | | |
| 96 | | | | | | | | |
| 97 | | | | | | | | |
| 98 | | | | | | | | |
| 99 | | | | | | | | |
| 100 | | | | | | | | |
| TOTAL | | | | | | | | |

Mean of A $= \frac{\Sigma x}{n} =$ Mean of C $= \frac{\Sigma x}{n} =$

Standard deviation $= \sqrt{\left(\frac{\Sigma d^2}{2n}\right)} =$ Standard deviation $= \sqrt{\left(\frac{\Sigma d^2}{2n}\right)} =$

$F = \frac{(\text{larger SD})^2}{(\text{smaller SD})^2} =$ ; significance

| | Test method | | | Comparative method | | |
|---|---|---|---|---|---|---|
| Low results | Mean = | SD = | $n =$ | Mean = | SD = | $n =$ |
| Medium results | Mean = | SD = | $n =$ | Mean = | SD = | $n =$ |
| High results | Mean = | SD = | $n =$ | Mean = | SD = | $n =$ |

*Figure 4 (b). Short evaluation summary.*

*Test method*

Supplier:
Batch or model No.:
Reagent(s):
Standard(s)

*Comparative method*

Supplier:
Batch or model No.:
Reagent(s):
Standard(s)

*Imprecision*

| | Test method | | | | Comparative method | | | |
|---|---|---|---|---|---|---|---|---|
| | *n* | *Mean* | *SD* | *CV* | *n* | *Mean* | *SD* | *CV* |
| Overall | | | | | | | | |
| Low results | | | | | | | | |
| Medium results | | | | | | | | |
| High results | | | | | | | | |

*Inaccuracy*

Regression equation:

Test method = × Comparative method ±

$r=$ $t=$ $n=$

THE FOLLOWING SECTIONS, IN CONJUNCTION WITH THE FOREGOING EXPERIMENT, CONSTITUTE A FULL EVALUATION

### (2) Between-day imprecision

*Materials*

At least three specimens are selected for analysis.

Great care must be taken to select materials (either pooled material from specimens from patients or quality control materials) with appropriate levels of analyte. It is recommended that:

(1) Where the analyte has a lower and upper medically significant decision level (for example plasma sodium) the low, medium and high materials should have analyte levels at the low decision level, mid-point of the reference interval and upper decision level.

(2) Where the analyte does not have a lower medically significant decision level (for example plasma bilirubin) the low, medium and high materials should have analyte levels at the upper limit of the reference at the decision level and near the extreme upper range capability of the method.

Liquid materials have a number of advantages, but, before use, should be subdivided into aliquots and separately frozen. Lyophilized material must be reconstituted using an identical protocol throughout the experiment, or, ideally, sufficient lyophilized material for the experiment should be reconstituted and pooled and then aliquots prepared and separately frozen.

The characteristics of the materials used should be recorded.

*Procedure*

Analyse one sample of each of the materials selected on each of 20 days or in 20 separate analytical batches spread over a number of working days. Ideally, the three or more materials should be analysed in purely random order amongst a run of specimens from patients; by this strategy the previously described within-run imprecision and inaccuracy experiment and assessment of between-day imprecision can be performed simultaneously. Ideally, a single batch of reagents should be used throughout this experiment. Where reagents are unstable, they should be prepared as required from the same lot of chemicals.

Record the results of the analyses in column A on the checklist; columns B and C may be used, if required, for calculations of standard deviation as described below.

*Data analysis*

The means and imprecisions, as standard deviation and coefficient of variation, are calculated. 99·8% confidence ranges are calculated as the mean ±3SD. Any results falling outside this range (outliers) are rejected. Further analyses, equal to the number of outliers, should be the performed and new means and imprecisions calculated.

*If calculation facilities are unavailable:*

(1) *Add all the results in column A.*
(2) *Divide this total by the number of analyses* ($n$) *to give the mean value* ($\bar{x}$).
(3) *Use column B to list values of result − mean* ($x-\bar{x}$).
(4) *Use column C to list values of* (*result − means*)$^2$ ($x-\bar{x}$)$^2$.
(5) *Add the values in column C to obtain* $\Sigma(x-x)^2$, *divide this by the number of analyses − 1* ($n-1$), *and take the square root of this number to obtain the standard deviation.*
(6) *Divide the standard deviation by the mean to obtain the coefficient of variation.*

Record the results of statistical analyses.

*Figure 4 (c). Between-day imprecision.*

*Materials*

*Low*

Type:
Supplier:
Batch No.:

*Medium*

Type:
Supplier:
Batch No.:

*High*

Type:
Supplier:
Batch No.:

*Between-day imprecision: Results for low material*

| *No.* | A *Result* | B (A − Mean) *Calculation* | C (A − Mean)$^2$ *Calculation* |
|---|---|---|---|
| 1 | | | |
| 2 | | | |
| 3 | | | |
| 4 | | | |
| 5 | | | |
| 6 | | | |
| 7 | | | |
| 8 | | | |
| 9 | | | |
| 10 | | | |
| 11 | | | |
| 12 | | | |
| 13 | | | |
| 14 | | | |
| 15 | | | |
| 16 | | | |
| 17 | | | |
| 18 | | | |
| 19 | | | |
| 20 | | | |
| Total | | | |

Mean ($\bar{x}$) = $\frac{\Sigma x}{n}=$

Standard deviation (SD) = $\sqrt{\left(\frac{\Sigma(x-\bar{x})^2}{n-1}\right)}=$

Coefficient of variation = $\frac{SD}{\bar{x}}=$

Mean ± 3 SD range is to

*continued —*

*Figure 4 (c). Between-day imprecision (continued).*

*Between-day imprecision: Results for medium material*

| No. | A *Result* | B (A − mean) *Calculation* | C (A − mean)$^2$ *Calculation* |
|---|---|---|---|
| 1 | | | |
| 2 | | | |
| 3 | | | |
| 4 | | | |
| 5 | | | |
| 6 | | | |
| 7 | | | |
| 8 | | | |
| 9 | | | |
| 10 | | | |
| 11 | | | |
| 12 | | | |
| 13 | | | |
| 14 | | | |
| 15 | | | |
| 16 | | | |
| 17 | | | |
| 18 | | | |
| 19 | | | |
| 20 | | | |
| Total | | | |

$$\text{Mean}(\bar{x}) = \frac{\Sigma x}{n} =$$

$$\text{Standard deviation (SD)} = \sqrt{\left(\frac{\Sigma(x-\bar{x})^2}{n-1}\right)} =$$

$$\text{Coefficient of variation} = \frac{\text{SD}}{\bar{x}} =$$

Mean ± 3 SD range is to

*Between-day imprecision: Results for high material*

| No. | A *Result* | B (A − mean) *Calculation* | C (A − mean)$^2$ *Calculation* |
|---|---|---|---|
| 1 | | | |
| 2 | | | |
| 3 | | | |
| 4 | | | |
| 5 | | | |
| 6 | | | |
| 7 | | | |
| 8 | | | |
| 9 | | | |
| 10 | | | |
| 11 | | | |
| 12 | | | |
| 13 | | | |
| 14 | | | |
| 15 | | | |
| 16 | | | |
| 17 | | | |
| 18 | | | |
| 19 | | | |
| 20 | | | |
| Total | | | |

$$\text{Mean}(\bar{x}) = \frac{\Sigma x}{n} =$$

$$\text{Standard deviation (SD)} = \sqrt{\left(\frac{\Sigma(x-\bar{x})^2}{n-1}\right)} =$$

$$\text{Coefficient of variation} = \frac{\text{SD}}{\bar{x}} =$$

Mean ± 3 SD range is to

*Graphical analysis*

In order to check for changes in inaccuracy during the experiment (drift) and to assess stability of the materials, all results are plotted as graphs.

The individual results obtained are plotted for each day of the experiment.

The scale of the 'results' axis should be chosen to be equivalent to the total range of results generated.

The graphs should be assessed visually for drift and/or instability. If this has occurred, then the experiment should be repeated taking corrective procedures such as selection of more stable materials or preparation of fresh reagents for each run.

### (3) Within-run imprecision

*Procedure*

One sample of each of the materials used in the evaluation of between-day imprecision is analysed 20 times in a *single* batch. If the instrument or reagent kit cannot handle this number of specimens in one analytical run, perform the maximum number of analyses possible. An alternative approach, which may be suitable for single or small vial reagent kits, is to reconstitute sufficient vials to provide reagent for 20 tests, pool the reagent and run the analyses.

Record the results of analysis as shown in figure 4 (*d*).

*Data analysis*

Calculate the means, standard deviations and coefficients of variation as described above. Check for outliers, reject as detailed above, and recalculate the statistical parameters. Record the results of statistical analysis.

*Figure 4 (d). Within-run imprecision.*

*Results for low material*

| No. | A *Result* | B (A − mean) *Calculation* | C (A − mean)$^2$ *Calculation* |
|---|---|---|---|
| 1 | | | |
| 2 | | | |
| 3 | | | |
| 4 | | | |
| 5 | | | |
| 6 | | | |
| 7 | | | |
| 8 | | | |
| 9 | | | |
| 10 | | | |
| 11 | | | |
| 12 | | | |
| 13 | | | |
| 14 | | | |
| 15 | | | |
| 16 | | | |
| 17 | | | |
| 18 | | | |
| 19 | | | |
| 20 | | | |
| Total | | | |

$$\text{Mean}(\bar{x}) = \frac{\Sigma x}{n} =$$

$$\text{Standard deviation (SD)} = \sqrt{\left(\frac{\Sigma(x-\bar{x})^2}{n-1}\right)} =$$

$$\text{Coefficient of variation} = \frac{\text{SD}}{\bar{x}} =$$

Mean ± 3 SD range is to

*continued*—

*Figure 4 (d). Between-day imprecision (continued).*

*Within-run imprecision: Results for medium material*

| *No.* | A<br>*Results* | B (A − mean)<br>*Calculation* | C $(A - \text{mean})^2$<br>*Calculation* |
|---|---|---|---|
| 1 | | | |
| 2 | | | |
| 3 | | | |
| 4 | | | |
| 5 | | | |
| 6 | | | |
| 7 | | | |
| 8 | | | |
| 9 | | | |
| 10 | | | |
| 11 | | | |
| 12 | | | |
| 13 | | | |
| 14 | | | |
| 15 | | | |
| 16 | | | |
| 17 | | | |
| 18 | | | |
| 19 | | | |
| 20 | ——— | ——— | ——— |
| Total | | | |

$$\text{Mean}(\bar{x}) = \frac{\Sigma x}{n} =$$

$$\text{Standard deviation (SD)} = \sqrt{\left(\frac{\Sigma(x-\bar{x})^2}{n-1}\right)} =$$

$$\text{Coefficient of variation} = \frac{\text{SD}}{\bar{x}} =$$

Mean ± 3 SD range is to

*Within-run imprecision: Results for high material*

| *No.* | A<br>*Result* | B (A − mean)<br>*Calculation* | C $(A - \text{mean})^2$<br>*Calculation* |
|---|---|---|---|
| 1 | | | |
| 2 | | | |
| 3 | | | |
| 4 | | | |
| 5 | | | |
| 6 | | | |
| 7 | | | |
| 8 | | | |
| 9 | | | |
| 10 | | | |
| 11 | | | |
| 12 | | | |
| 13 | | | |
| 14 | | | |
| 15 | | | |
| 16 | | | |
| 17 | | | |
| 18 | | | |
| 19 | | | |
| 20 | ——— | ——— | ——— |
| Total | | | |

$$\text{Mean}(\bar{x}) = \frac{\Sigma x}{n} =$$

$$\text{Standard deviation (SD)} = \sqrt{\left(\frac{\Sigma(x-\bar{x})^2}{n-1}\right)} =$$

$$\text{Coefficient of variation} = \frac{\text{SD}}{\bar{x}} =$$

Mean ± 3 SD range is to

### (4) *Assessment of inaccuracy using control materials*

Commercially available control materials or calibration materials may have assigned values. These assigned values may be inaccurate because foreign materials and non-human materials have been added during manufacture and the physical and chemical properties may not truly reflect those of specimens from patients. However, if materials from a variety of sources give results by the test method that differ from those assigned, then that method is unlikely to have a satisfactory standard of accuracy. Materials which have been used in inter-laboratory quality assurance schemes (and therefore have had a consensus value for an analyte derived from many laboratories) are particularly useful for this part of the evaluation. Ideally, a range of materials should be selected that represent the various types of base material currently used and contain different levels of analyte.

### *Procedure*

Analyse at least six different materials in triplicate. These replicate analyses are performed in different analytical batches. The results are recorded as shown in figure 4 (*e*). The mean of the set of three results is calculated and recorded and the mean compared to the assigned or consensus value. For each mean result, calculate the allowable error using the formula:

$$\text{Allowable error} = \pm \frac{2 \times \text{SD}}{\sqrt{\text{No. of replicates}}} = \pm 1{\cdot}2 \times \text{SD}.$$

The between-day SD appropriate to that level of analyte is used in this formula.

Record the allowable error and an overall assessment of all results.

### (5) *Evaluation of linearity*

Many methods use a simple one-point or two-point calibration or standardization technique. The assumptions are then made that the method is linear at least between the origin and the level of the one-point calibration or between the two levels of a two-point calibration. Since good linearity is usually a necessary prerequisite for acceptable inaccuracy, it must be objectively assessed and not assumed.

### *Procedure*

Pipette into labelled tubes, in duplicate, samples of a specimen from a patient; the specimen should be selected to have a high level of analyte. Suitable volumes are:

0, 0·2, 0·4, 0·6, 0·8 and 1·0 ml (or multiples thereof).

To one set of tubes add:

1·0, 0·8, 0·6, 0·4, 0·2 and 0 ml (or appropriate multiples)

of distilled water, physiological saline or appropriate diluent, for example hormone-depleted serum for immunoassay techniques. To the other set of tubes, add:

1·0, 0·8, 0·6, 0·4, 0·2 and 0 ml (or appropriate multiples)

of a specimen from a patient which has a low level of analyte. Note the volumes used as shown in figure 4 (*f*).

Analyse both sets of samples in duplicate and insert the results as indicated in figure 4 (*f*). The duplicate analyses should be performed in *different* analytical batches.

### *Graphical analysis and calculations*

On graph paper, plot the mean result obtained for each sample against the volume of high sample used

Inspect the plots visually and draw the best straight line through the points.

*Figure 4(e). Analysis of control materials.*

| | Material | Results | Mean | Error | Assigned Value |
|---|---|---|---|---|---|
| Type: | | 1 | | | |
| Supplier: | | 2 | | | |
| Batch No.: | | 3 | | | |
| Type: | | 1 | | | |
| Supplier: | | 2 | | | |
| Batch No.: | | 3 | | | |
| Type: | | 1 | | | |
| Supplier: | | 2 | | | |
| Batch No.: | | 3 | | | |
| Type: | | 1 | | | |
| Supplier: | | 2 | | | |
| Batch No.: | | 3 | | | |
| Type: | | 1 | | | |
| Supplier: | | 2 | | | |
| Batch No.: | | 3 | | | |
| Type: | | 1 | | | |
| Supplier: | | 2 | | | |
| Batch No.: | | 3 | | | |

*Figure 4(f). Linearity.*

*High sample diluted with water/saline/other*

| No. | Vol. sample | Vol. diluent | Result | Result | Mean | Error |
|---|---|---|---|---|---|---|

*High sample diluted with low sample*

| No. | Vol. high sample | Vol. low sample | Result | Result | Mean | Error |
|---|---|---|---|---|---|---|

In order to further assess linearity in a more objective manner, find the between-day imprecision appropriate for each specimen analysed. Calculate the allowable error for each specimen analysed using the formula:

$$\text{Allowable error} = \pm \frac{2 \times \text{SD}}{\sqrt{\text{No. of replicates}}} = \pm 1{\cdot}4 \times \text{SD}$$

and record the appropriate allowable errors as shown in figure 4(*f*).

Assess whether the plotted point for each mean of duplicate analyses differs from the line of best fit by less than the allowable error: the method is linear where this criterion is fulfilled. Record the overall assessment of the linearity.

### (6) *Recovery*

An additional aid in the assessment of inaccuracy is to measure the amount of analyte in a sample following the addition of a known amount of pure analyte. Such recovery experiments are particularly useful in the evaluation of analytical methods in which significant losses may occur, for example, extraction procedures or chromatographic analysis.

Recovery experiments may be very difficult to design if the analyte is not available or exists only in a non-physiological form. In addition, since analyte is added to material already containing the analyte, analyses may be required to be performed at the upper extreme of the method's analytical range. All recovery experiments require some type of addition of material which may well change the matrix in subtle and undefined ways. Similarly, treatment of specimens from patients prior to addition of analyte, for example charcoal stripping to remove hormones, may markedly alter the nature of the matrix. Therefore, recovery experiments should only be attempted if there is confidence that the matrix is minimally affected by any addition.

### *Procedure*

A number of specimens from patients can be combined to provide a base pool. Ideally, the pure analyte is weighed directly into a small volume of the base pool in a volumetric flask and the volume made up to the mark with the base pool. Alternatively,

*Figure 4 (g). Recovery.*

Pure analyte:
Supplier:
Batch No.:
Form added:

| A *Sample* | B *Results* | C *Mean* *P* | D *Recovery* *C−P* | E *Expected* | F *Percentage* $\frac{C-P}{E} \times 100\%$ |
|---|---|---|---|---|---|
| Pool | 1. 2. | | | | |
| Pool+ | 1. 2. | | | | |
| Pool+ | 1. 2. | | | | |
| Pool+ | 1. 2. | | | | |

the analyte can be dissolved in a small amount of a suitable solvent. Aliquots of either of these preparations are added to aliquots of the base pool and the resulting samples and the base pool are analysed, in at least duplicate, in different analytical batches. At least three samples should be assessed. The pure analyte should be added to raise the level of the base sample by approximately 20%, 50% and 100%. The amounts added should not cause the total analyte concentration to exceed the upper range limit of the method.

The data from the analyses are recorded as indicated in figure 4 (*g*).

*Data analysis*
The amount added to aliquots of the base pool are recorded in column A, results obtained are recorded in column B and the mean of the duplicate analyses calculated and recorded in column C. Subtract the mean result for the base pool from the means of all other results and insert the data in column D. List the level of analyte expected to be recovered (the amount added, adjusted for volume if necessary) in column E and calculate the percentage recovery as the amount found divided by the amount added × 100, that is:

$$\text{percentage recovery} = \frac{\text{value in column D}}{\text{value in column E}} \times 100.$$

Calculate and record the mean recovery.

### (7) *Specificity and interference*

Inaccurate results may occur because constituents present in samples react and contribute to the final reading: this is described as *lack of specificity*. For example acetoacetate, which may occur at high levels in plasma specimens from diabetic patients, reacts with the alkaline picrate reagent used for creatinine determination to give falsely high results. *Interference* occurs when constituents in samples do not react in the method themselves but produce low results (inhibition) or high results (enhancement). For example, *enhancement* by haemoglobin may occur in any colorimetric method where measurements of absorbance are made in the region at which haemoglobin itself absorbs.

Careful review of the chemical and/or physical principles of the test method can identify constituents that may potentially cause problems. It is a difficult task to test for nonspecificity and interference since any drug, nutrient or constituent must be considered as having the potential to cause inaccuracy until it has been experimentally proved to have no effect.

The vast range of drugs in current use poses particular problems. Drugs may interfere in the chemistry of the method (for example prednisolone may give falsely elevated results with plasma cortisol reagent kit sets), or may cause physiological changes which affect laboratory tests (for example oral contraceptives can cause elevated plasma thyroid hormone or cortisol concentrations by raising the respective plasma-binding protein levels). The former type of effect can be simply investigated; experiments can be performed in a manner similar to those detailed here,that is addition of drug to specimens from patients with known levels of analyte and subsequent re-assay. The literature on known drug interferences should certainly be consulted (see Bibliography in section X).

It is recommended, as an absolute minimum, that the effects of bilirubin, haemoglobin and lipaemia be investigated.

*Procedure*
Prepare a base pool of specimens from patients which are not icteric, haemolysed or lipaemic. Select further specimens that have significant icterus, haemolysis and lipaemia. The true level of the analyte in these latter samples must be assessed. Therefore, analyse these by the *comparative method* and record the results as shown in figure 4 (*h*). If the comparative method is specific, then the true values for the analyte are found. If, in contrast, the comparative method suffers from lack of specificity, alternative approaches are:

(1) Analysis of the specimens in another laboratory which has a suitable 'reference' method, for example analysis of lipaemic samples for sodium could be carried out by direct ion-selective electrode technology.

*Figure 4 (h). Specificity and interference.*

*Icterus*

| | Base pool = | | Icteric sample = | Bilirubin = | | |
|---|---|---|---|---|---|---|
| *No.* | *Vol. pool (A)* | *Vol. sample (B)* | *Bilirubin (C)* | *Calc. result (D)* | *Found (mean) (E)* | *Interference (F)* |

*Haemolysis*

| | Base pool = | | Haemolyzed sample = | | Haemoglobin = | |
|---|---|---|---|---|---|---|
| *No.* | *Vol. pool (A)* | *Vol. sample (B)* | *Haemoglobin (C)* | *Calc. result (D)* | *Found (mean) (E)* | *Interference (F)* |

*Lipaemia*

| | Base pool = | | Lipaemic sample = | | Triglycerides = | |
|---|---|---|---|---|---|---|
| *No.* | *Vol. pool (A)* | *Vol. sample (B)* | *Triglycerides (C)* | *Calc. result (D)* | *Found (mean) (E)* | *Interference (F)* |

(2) Analysis of samples of base pool with known values of bilirubin and haemoglobin which have been generated by addition of appropriate volumes of specially prepared solutions of bilirubin and haemoglibin to aliquots of the base pool.

Analyse the three samples for bilirubin, haemoglobin and triglycerides respectively, using the best methods available in the routine laboratory, and record the results as indicated in figure 4 (*h*). Prepare three series of aliquots of the base pool in labelled tubes.

Suitable volumes are:

0, 0·2, 0·4, 0·6, 0·8 and 1·0 ml (or multiples thereof).

To these samples, add:

1, 0, 0·8, 0·6, 0·4, 0·2 and 0 ml (or appropriate multiples)

of the icteric, haemolysed and lipaemic samples to generate three series of samples with linearly increasing levels of icterus, haemolysis and lipaemia respectively. Analyse these samples, at least in duplicate, in separate batches and record the mean of the results as shown in figure 4 (*h*).

*Data analysis*

From the relative volumes of base pool (column A) and sample with potentially interfering constituent (column B), calculate the expected results and record in column D. Subtract these results from the results found and record the interference in column F. From the level of potentially interfering constituent (which should be calculated and recorded in column C), examine whether the interference is directly proportional to this level. If the interference is directly proportional, calculate the effect as units of test method analyte per unit of interfering constituent. Record the overall interferences found.

### *(8) Detection limit*

The detection limit is the smallest single result which can be distinguished from a true blank and should be used as the practical lower limit of the measurement range.

*Procedure*

Select at least five samples which are suitable blanks. A true blank is the matrix that is devoid of the analyte assayed by the test method. If true blanks are unavailable, they can be simulated by omission of a crucial reagent or a critical part of the analytical procedure (for example incubation). Analyse these samples in duplicate and record the results as shown in figure 4 (*i*). Since the imprecision of the method may have been significantly changed by this analytical modification the imprecision of the method at very low levels of analyte should be measured. Carefully select at least five samples which have very low levels of analyte and which therefore will have readings close to the range given by the true or simulated blanks. Analyse these samples in duplicate and record the results as indicated in figure 4 (*i*).

*Data analysis*

Calculate the mean and standard deviation of the true or simulated blanks from the formulae:

$$\bar{x} = \frac{\Sigma x}{n} \qquad SD = \sqrt{\left(\frac{\Sigma d^2}{2n}\right)}.$$

Similarly calculate the standard deviation of the samples with the low levels of analyte. The two standard deviations should be similar; this hypothesis can be tested using the F-test. If the standard deviations for the blanks and the low level

*Figure 4 (i). Detection limit.*

*True blanks*

| | *No.* | *Result 1* | *Result 2* | *Calculation (d)* | *Calculation ($d^2$)* |
|---|---|---|---|---|---|
| | | | | | |
| Totals | | | | | |

No. of pairs ($n$) =

$$\text{mean } (\bar{x}) = \frac{\Sigma x}{n} =$$

$$SD = \sqrt{\left(\frac{\Sigma d^2}{2n}\right)} =$$

*Low samples*

| | *No.* | *Result 1* | *Result 2* | *Calculation (d)* | *Calculation ($d^2$)* |
|---|---|---|---|---|---|
| | | | | | |
| Totals | | | | | |

No. of pairs ($n$) =

$$\text{mean } (\bar{x}) = \frac{\Sigma x}{n} =$$

$$SD = \sqrt{\left(\frac{\Sigma d^2}{2n}\right)} =$$

*Figure 5. Full evaluation summary.*

Re-evaluate data from short evaluation: figure 4 (*b*).

*Imprecision*

| | *Between-day* | | | | *Within-run* | | | |
|---|---|---|---|---|---|---|---|---|
| | *n* | *Mean* | *SD* | *CV* | *n* | *Mean* | *SD* | *CV* |
| Low | | | | | | | | |
| Medium | | | | | | | | |
| High | | | | | | | | |

*Quality control materials*

Are the assigned values obtained?:
Are there problems or potential problems with materials?:

*Recovery*

Mean recovery:

*Linearity*

Upper limit of linearity
(sample diluted with water/saline/diluent):
Upper limit of linearity
(high sample diluted with low sample):

*Specificity and interference*

| | | |
|---|---|---|
| Interference with icterus: | per | bilirubin |
| Interference with haemolysis: | per | haemoglobin |
| Interference with lipaemia: | per | triglycerides |

*Detection limit*

Detection limit:

analyses are similar the detection limit may then be calculated from the approximate formula:

$$\text{Detection limit} = \text{mean of true blank} \times 2{\cdot}8 \times \text{SD}.$$

Record the detection limit.

If the standard deviations are significantly different, other methods of simulating true blanks will have to be found.

### Section VII: Specific studies

Section VI detailed an evaluation protocol that is applicable to all methods, instruments and reagent kit sets. However, most methods possess features that are unique to themselves and clearly these singular aspects should also be subject to rigorous evaluation. Therefore *specific studies* form the second major experimental component of a complete evaluation.

The aim of these specific studies is to detect, in the test method, any problems that exist or may arise with:

(1) Component performance.
(2) Method chemistry.

It is important to realize that these specific studies must be carried out regardless of whether the test method performed well or poorly in the Section VI evaluation. Enduring good performance will be dependent upon the individual components continuing to function to the same standards reached during the evaluation. An unacceptable outcome in Section VI may be traceable either to components that comprise the test method itself, or to components that have to be supplied by the laboratory. In either case the components, for example pipettors, radioactivity counters and colorimeters, must be individually evaluated to ensure that they function optimally and will continue to do so after the complete evaluation is concluded.

A general evaluation cannot account for all the types of specimen that the test method may be called on to analyse. Therefore, specific studies should be designed to explore the effects of potential samples on the chemistry of the test method. Such studies should pay particular attention to differences between human and animal sera, since many quality control and assurance sera use the latter matrix.

Clearly, it is not possible to detail protocols that will embrace the special studies required for the range of currently available instruments and reagent kit sets. Therefore those areas that are strongly recommended as candidates for specific studies are briefly listed below. The Bibliography (section X) should be consulted if guidance about individual experimental design is required.

*Spectrophotometers:*
Wavelength range
Band width
False light
Temperature control
Linearity
Inaccuracy and imprecision
Warm-up time.

*Mechanized analysers:*
Carry-over
Specimen-cross contamination
Specimen-diluent contamination
Drift
Reagent usage
Automatic calculation of results from raw data.

*Incubators:*
Temperature stability
Warm-up time.

*Diluters/dispensers/samplers:*
Imprecision and inaccuracy
Specimen-cross contamination
Specimen-diluent contamination.

*Enzyme analysers:*
Achievement of true end-point
Optimal conditions
Cofactor concentration
Analyte level at which substrate depletion occurs.

*Reagents and reagent kit sets:*
Labelling requirements
Stability and shelf-life.

*Samples:*
Diluted patient specimens
Bovine and other animal quality control materials.

*Ligand assay reagent kit sets:*
Standards in aqueous solution, bovine, or human sera
Effect of total protein concentration on separation procedures
Effect of small variations in timing of procedures, such as incubation and separation
Effect of small variations in temperature on procedures
Dilution of sample to parallel standard curve
Cross-reactivity
Time to obtain an adequate number of counts.

*Scintillation counters:*
Calibration
Efficiency
Effect of sample size and position
Background
Line-voltage stability
Channel width
Dector equivalence in multi-well counter
Linearity
Efficiency of scintillation cocktails.

*Data reduction:*
Errors of manual and automated calculation of results.

*Balances:*
Inaccuracy and imprecision.

*Manual tests:*
Adaptability to mechanization or automation.

### Section VIII: Acceptability criteria

It is a difficult task to decide whether the test method is acceptable: there is little published work to aid in this decision. A logical sequential flow-diagram is presented in figure 6 to help decision-making.

Criteria for acceptance or rejection of the method are as follows.

### *(1) Within-run imprecision*

After outliers have been rejected on objective grounds, the within-run imprecision of the method can be accepted as satisfactory if the SD is equal to or less than half of the intra-individual biological variation of the analyte (the ideal), or

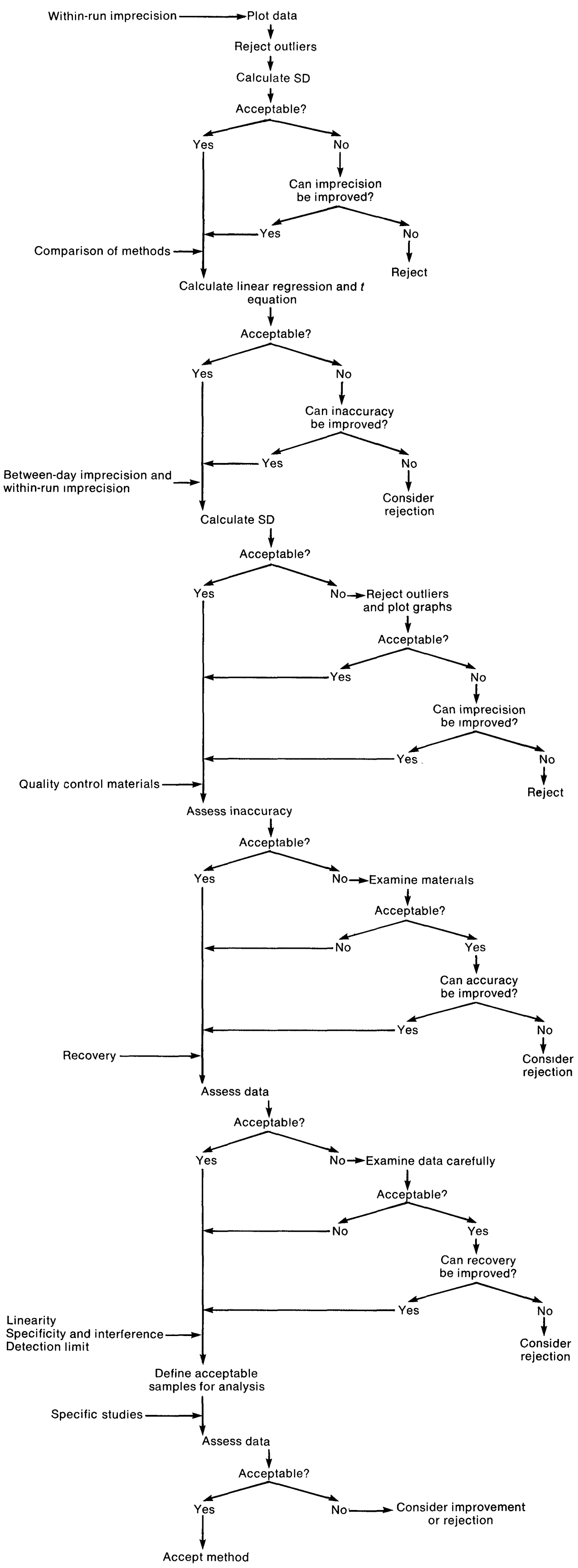

*Figure 6. Logical sequential flow diagram for acceptability criteria.*

fulfills another satisfactory analytical goal for imprecision (see Bibliography in Section X). If the SD is greater than the goal, assess whether the method can be improved by, for example, introduction of replicate analyses or modification of the type and level of standard. If the method cannot be improved, reject it.

### (2) *Comparison of methods*

Interpretation of the statistics used for comparison of methods data is particularly difficult, especially if the comparative method is far from ideal itself (see Bibliography in Section X). The test method can be accepted:

(*a*) If *r* is greater than 0·99.
(*b*) If the slope is not significantly different from 1·00.
(*c*) If the intercept is not significantly different from 0.
(*d*) If *t* shows that the means are not different.

If these criteria are not fulfilled, assess whether the method can be improved by, for example, use of different standards. If the method cannot be improved, seriously consider rejection unless acceptance can be fully justified on objective clinical grounds.

### (3) *Between-run and within-run imprecision*

The criteria adopted for within-run imprecision should also be applied here.

### (4) *Quality control materials*

If the results obtained by the test method are not significantly different from the assigned values, that is the assigned value lies within the result ± allowable error, accept the method. If the results are different, examine whether the quality control materials themselves pose problems that do not occur with specimens from patients, for example turbidity caused by lyophilization. If the materials are suspect, accept the method. If the materials are not suspect, assess whether the accuracy of the method can be improved. If the method cannot be improved, seriously consider rejection unless acceptance can be fully justified on objective clinical grounds.

### (5) *Recovery*

If the mean recovery is 90–110%, and no individual recovery is less than 85% or more than 115%, accept the method. If these criteria are not fulfilled, re-examine the design of the recovery studies. If the experiment could only be performed in a non-ideal manner, for example if iron was added as a solution of iron metal in hydrochloric acid and significant pH changes subsequently occurred, the results can be regarded with scepticism as to their validity.

### (6) *Specific studies*

Assess data derived from all specific studies. If the results are satisfactory, accept the method. If the results are not satisfactory, give careful consideration as to whether an improvement is technically possible at reasonable cost. Otherwise, consider rejection of the method.

### (7) *Method acceptance and definition of samples*

The test method is acceptable if all the above criteria are fulfilled. Careful assessment of linearity, specificity and interference, and detection limit should be undertaken to define those samples that are not suitable for analysis by the method.

### Section IX: Introduction to service

If the evaluation, specific studies and the final assessment of performance has led to an objective decision to accept the test method, then the method is ready to be introduced into routine service. Before the method finally becomes operational in the routine setting there are a number of important ancillary tasks to be completed.

(1) Preparation of work site if evaluation has been performed in a special area.
(2) Training of staff in the operation and performance of the test method, maintenance, and trouble-shooting; education of staff on the principles of the method and its chemistry; guidelines for emergency and out-of-hours use and associated trouble-shooting.
(3) Preparation of work-lists, quality control and decision criteria for monitoring of performance, interfaces if online data reduction is envisaged and reporting systems.
(4) Purchase of sufficient reagents, quality control materials, and spares and consumables and organization of storage. Assessment of reliability of manufacturer or agent to provide standing orders or regular delivery.
(5) Determination of reference interval if the evaluation studies revealed that the new method requires a reference interval that is significantly different from current usage.
(6) Preparation of a routine maintenance schedule and associated check-list.
(7) Preparation of an insert for the laboratory method record, detailing clinical use of method, method principle, source of method, specimen collection requirements, standards, quality control, reagent composition, procedure, result units, and relevant cautions (effects of lipaemia, haemolysis, drugs etc.).
(8) Enrolment of the method in an external quality assurance scheme.
(9) Communication with all laboratory users if the introduction of the new method will alter specimen requirements, reference intervals or turnaround time of results.

Although the new method has now been fully evaluated and installed into the routine laboratory the completed 'Evaluation Kit' should not be discarded. The data contained in it comprise as full an objective description of the new method that it is usually possible to have, specifying how the method actually did perform in all relevant aspects. Therefore the 'Evaluation Kit' provides the bench-mark against which all future quality control and assurance programme results can be compared.

### Section X: Bibliography

*Terminology*

Buttner, J., Borth, R., Boutwell, J. H., Broughton, P. M. G. and Bowyer, R. C., Approved recommendation (1978) on quality control in clinical chemistry. Part 1. General principles and terminology. *Clinica Chimica Acta*, **98** (1979), 129F–143F.

NCCLS Proposed Standard: PSC-13. *Nomenclature and Definitions for Use in the National Reference System in Clinical Chemistry* (National Committee for Clinical Laboratory Standards, Villanova, Pennsylvania, 1979).

*Statistics*

Barnett, R. N., *Clinical Laboratory Statistics* (Little, Brown and Co., Boston, Massachusetts, 1971).

Westgard, J. O. and Hunt, M. R., Use and interpretation of common statistical tests in method comparison studies. *Clinical Chemistry*, **19** (1973), 49–57.

*Evaluation protocols*

Westgard, J. O., Precision and accuracy: concepts and assessment by method evaluation testing. *CRC Critical Reviews in Clinical Laboratory Sciences*, **14** (1981), 283–330.

Buttner, J., Borth, R., Boutwell, J. H., Broughton, P. M. G. and Bowyer, R. C., Approved recommendation (1978) on quality control in clinical chemistry. Part 2. Assessment of analytical methods for routine use. *Clinica Chimica Acta*, **98** (1979), 145F–162F.

Percy-Robb, I. W., Broughton, P. M. G., Jennings, R. D., McCormack, J. J., Neil, D. W., Saunders, R. A. and Warner, M., A recommended scheme for the evaluation of kits in the clinical chemistry laboratory. *Annals of Clinical Biochemistry*, **17** (1980), 217–226.

Broughton, P. M. G. Evaluation of analytical methods in clinical chemistry. *Progress in Clinical Pathology*, **7** (1979), 1–31.

Kim, E. K. and Logan, J. E., A scheme for the evaluation of methods in clinical chemistry with particular application to those measuring enzyme activities. Part 1. General considerations. *Clinical Biochemistry*, **11** (1978), 238–243.

NCCLS Proposed Standards: PSEP-2, PSEP-3, and PSEP-4. *Protocol for Establishing Performance Claims for Clinical Chemical Methods. Introduction and Performance Check Experiment, Replication Experiment, and Comparison of Methods Experiment* (National Committee for Clinical Laboratory Standards, Villanova, Pennsylvania, 1979).

*Reference and definitive methods*

Tietz, N. W., A model for a comprehensive measurement system in clinical chemistry. *Clinical Chemistry*, **25** (1979), 833–835.

*Quality-control materials*

Fraser, C. G. and Peake, M. J., Problems associated with clinical chemistry quality control materials. *CRC Critical Reviews in Clinical Laboratory Sciences*, **12** (1980), 59–86.

*Drug interferences*

Young, D. S., Pestaner, L. C. and Gibberman, V., Effect of drugs on clinical laboratory tests. *Clinical Chemistry*, **21** (1975), 1D–432D.

*Acceptable performance standards*

Fraser, C. G., Analytical goals in clinical biochemistry. *Progress in Clinical Pathology*, **8** (1981), 101–122.

Fraser, C. G., Desirable performance standards for clinical chemistry tests. *Advances in Clinical Chemistry*, **23** (1983), 299–339.

*Biological variation*

Van Steirteghem, A. C., Robertson, E. A. and Young, D. S., Variance components of serum constituents in healthy individuals. *Clinical Chemistry*, **24** (1978), 212–222.

Shephard, M. D. S., Penberthy, L. A. and Fraser, C. G., Short- and long-term biological variation in analytes in urine of apparently healthy individuals. *Clinical Chemistry*, **27** (1981), 569–573.

*Data reduction*

Challand, G. S., Automated calculation of radioimmunoassay results. *Annals of Clinical Biochemistry*, **15** (1978), 123–135.

Jeffcoate, S. L. and Das, R. E. G., Interlaboratory comparison of radioimmunoassay results. *Annals of Clinical Biochemistry*, **14** (1977), 258–260.

